# Data-Independent Acquisition for the Quantification and Identification of Metabolites in Plasma

**DOI:** 10.3390/metabo10120514

**Published:** 2020-12-18

**Authors:** Tom van der Laan, Isabelle Boom, Joshua Maliepaard, Anne-Charlotte Dubbelman, Amy C. Harms, Thomas Hankemeier

**Affiliations:** Analytical Biosciences and Metabolomics, Division of Systems Biomedicine and Pharmacology, Leiden Academic Center for Drug Research, Leiden University, 2333 CC Leiden, The Netherlands; t.van.der.laan@lacdr.leidenuniv.nl (T.v.d.L.); isabelleb@hotmail.nl (I.B.); joshuamaliepaard@hotmail.com (J.M.); a.c.dubbelman@lacdr.leidenuniv.nl (A.-C.D.); a.c.harms@lacdr.leidenuniv.nl (A.C.H.)

**Keywords:** data-independent acquisition, SWATH, MS^ALL^, mass spectrometry, metabolomics, chromatography

## Abstract

A popular fragmentation technique for non-targeted analysis is called data-independent acquisition (DIA), because it provides fragmentation data for all analytes in a specific mass range. In this work, we demonstrated the strengths and weaknesses of DIA. Two types of chromatography (fractionation/3 min and hydrophilic interaction liquid chromatography (HILIC)/18 min) and three DIA protocols (variable sequential window acquisition of all theoretical mass spectra (SWATH), fixed SWATH and MS^ALL^) were used to evaluate the performance of DIA. Our results show that fast chromatography and MS^ALL^ often results in product ion overlap and complex MS/MS spectra, which reduces the quantitative and qualitative power of these DIA protocols. The combination of SWATH and HILIC allowed for the correct identification of 20 metabolites using the NIST library. After SWATH window customization (i.e., variable SWATH), we were able to quantify ten structural isomers with a mean accuracy of 103% (91–113%). The robustness of the variable SWATH and HILIC method was demonstrated by the accurate quantification of these structural isomers in 10 highly diverse blood samples. Since the combination of variable SWATH and HILIC results in good quantitative and qualitative fragmentation data, it is promising for both targeted and untargeted platforms. This should decrease the number of platforms needed in metabolomics and increase the value of a single analysis.

## 1. Introduction

Fluctuations in the levels of metabolites have frequently been correlated to health, disease or response to treatment [1,2,3]. Therefore, the identity and quantity of these metabolites can help researchers and clinicians to determine the health status of an individual and eventually to realize personalized medicine [4]. Liquid chromatography (LC) coupled to mass spectrometry (MS) is one of the most commonly used techniques to identify and quantify metabolites in biological samples by qualitative and quantitative analyses, respectively [5,6]. This is due to its high sensitivity, selectivity and potential for high throughput.

With high-resolution MS, a full MS scan can be used to suggest the elemental composition of a compound based on the measured *m*/*z* value. However, the intact mass alone does not provide enough structural information to identify unknown features or resolve compounds with the same *m*/*z* value, i.e., isomers. Fragmentation patterns obtained by tandem mass spectrometry (MS/MS) provide structural information and can, therefore, aid in identifying metabolites and resolving isomers. Therefore, fragmentation data are a great asset in both qualitative and quantitative analyses.

MS/MS can be subdivided into two categories: targeted and untargeted analyses, whereby the latter can be further subdivided into data-dependent (DDA) and data-independent acquisition (DIA) protocols. An overview of the most commonly used MS/MS modes is listed in Table 1. In targeted analyses, one or multiple metabolites are selected based on the hypothesis of a biological question. Most often, these analyses are performed by multiple reaction monitoring (MRM) using a triple quadrupole (QqQ) mass spectrometer in which a precursor and product ion(s) for each analyte are optimized [7]. A more selective and comprehensive targeted approach can be realized by MRM high-resolution (MRM^HR^) (also known as parallel reaction monitoring (PRM)). In this mass spectrometry mode, a preselected precursor is isolated and fragmented followed by a high-resolution scan of all the produced product ions. Targeted analyses only allow for the quantification and identification of preselected features. This means that it is possible to optimize analytical parameters specifically to an analyte, which can be beneficial in terms of selectivity and sensitivity. It also leads to less unnecessary data, because of the preselection of interesting targets.

Untargeted analysis aims at a comprehensive measurement of all analytes in a biological system, including chemical unknowns [8,9]. MS/MS scans are indispensable in untargeted analysis to provide structural information. Untargeted analyses, i.e., DDA- and DIA-based protocols, consist of a high-resolution full scan followed by a single or multiple MS/MS experiment(s). In DDA, precursor ions are usually selected and fragmented based on intensity resulting in the fragmentation of the most abundant precursor ions [10]. A major drawback of these methodologies is the limited time for fragmentation, which is caused by the peak width and MS scan time. Time limits the fragmentation to the most abundant precursor ions, which can result in missing fragmentation data for low abundant ions. This can be partly overcome by including a dynamic exclusion list, e.g., based on time beyond the top N intensity peaks. Excluding already fragmented *m*/*z* values for a certain amount of time will increase the fragmentation range of a DDA analysis. However, the fact remains that this type of analysis is naturally biased towards certain analytes [11]. Chromatograms acquired through DDA cannot be used for quantification, because often, the apex of the peak is missed, and/or there are not enough data points on the peak for accurate quantitation [12].

DIA fragmentation is not triggered by the intensity of precursor ions but is predefined by the user. Here, a certain mass range is selected, and all precursors within this mass range are fragmented. Since DIA methodologies do not involve a selection of precursor (apart from the selected mass range), they are often considered more suitable for untargeted analyses [8,9,13]. One example of DIA is MS^ALL^ (also known as all-ion fragmentation (AIF) and MS^E^) in which the whole mass range is fragmented at once [14,15]. This technique makes use of a wide Q1 (first quadrupole) filter and allows for the detection of the fragments of all precursors in a single scan. A more selective DIA approach is called sequential window acquisition of all theoretical mass spectra (SWATH) in which a mass range of interest is divided into several subranges [13]. Typically, SWATH scans are performed by Q1 window sizes ranging from 3–50 Dalton. In proteomics, this window size is typically 25 Dalton [16]. Since the DIA analyses allow for the fragmentation of all precursors, the quality of DIA acquired MS/MS spectra might be compromised by the larger Q1 window size. More precursors will enter the collision cell concurrently with increasing Q1 window sizes. As a result, complex MS/MS spectra can hamper the proper identification of chemical unknowns, and overlap in product ion formation can lead to inaccurate quantification. [8] This is especially problematic for high-throughput analyses whereby numerous analytes are coeluting. Moreover, fast analyses often go hand in hand with a narrow chromatographic peak width resulting in less time for fragmentation.

The fragmentation data of DIA methodologies can be used for two purposes: identification and quantification. This offers great potential for combined quantitative and qualitative platforms, which are increasingly implemented in the field of metabolomics [17]. For the identification of metabolites, the acquired MS/MS spectra can be compared with commercially available spectral libraries, such as NIST, mzCloud, the Human Metabolome Database (HMDB) and Metlin. These libraries are easily searched in open access web interfaces and/or by the integration into data analysis software. Metabolite identifications can be scored based on the similarity in fragmentation patterns between the acquired spectra and library spectra. Other identification criteria include isotope peak intensity ratios, peak-to-peak Pearson correlation (PPC) and peak-to-peak shape (PPS) ratios, for example [18,19]. It is also possible to re-interrogate the data for the identification of unknown features found at a later stage, since MS/MS spectra of all precursors are acquired. The human metabolome consists of numerous metabolites, which makes its composition very complex. The Human Serum Metabolome, for example, reports the presence of 4651 metabolites [20]. Because of the complexity of the human metabolome, the use of comprehensive fragmentation techniques is as important as the use of comprehensive libraries. Current spectral libraries cover a substantial part of the human metabolome (e.g., 3500 human metabolites in NIST 2017 [21] and 3724 endogenous metabolites in mzCloud [22]). This coverage is essential in identifying unknown metabolites using MS/MS data.

For quantification, fragmentation can help in quantifying compounds that are not resolved by their exact mass, like isomers. Chromatographic separation is often used to resolve isomers before introducing them into the MS. However, achieving baseline separation between isomers often requires extensive method development and these analytical platforms are usually targeted to only a single isomer pair [23,24]. Generally, DIA platforms are not optimized to resolve specific isomer pairs. The fragmentation data in DIA analyses, however, can be used to differentiate between structural isomers, which have the same elemental composition but differ in elemental organization. As a result, different fragmentation patterns can be obtained within a structural isomer pair [25,26]. Diagnostic product ions can be used to differentiate between structural isomers and can also help to quantify these compounds individually.

In this work, we evaluate the versatility of DIA methodologies for metabolomics. Both a conventional LC-MS and a high-throughput platform were evaluated based on their compatibility with DIA analyses. The high-throughput platform consisted of a fractionation-based separation using three short chromatographic columns and an analysis time of 3 min [27]. These platforms differ in separation efficiency and peak width and thereby reflect the demands of different types of chromatography in terms of DIA analyses. The compatibility assessment is based on the qualitative and quantitative performances of the MS/MS scans. In order to demonstrate the quantitative performance, we have quantified five structural isomer pairs by SWATH and MS^ALL^ using diagnostic product ions. The quantified values are compared with MRM^HR^ for accuracy, since this is the most selective fragmentation mode. The qualitative performance is defined by identifying a set of known metabolites using a commercially available MS/MS library (NIST). This comprehensive comparison of different DIA methodologies using different separation mechanisms should demonstrate the usefulness and the limitations of DIA methodologies in terms of quantification and identification.

## 2. Results and Discussion

### 2.1. Method Development

**Figure 1:**
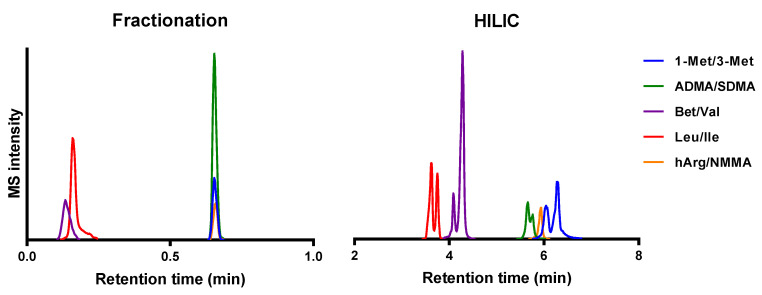
Extracted ion chromatograms of five structural isomer pairs measured in positive ionization mode. The fractionation method does not demonstrate chromatographic separation within an isomer pair. The hydrophilic interaction liquid chromatography (HILIC) method does demonstrate slight chromatographic separation within an isomer pair.

For the assessment of the DIA performance, we have chosen two different chromatography methods to demonstrate the possibilities and limitations of DIA-based analyses. The difference between the two chromatography methods is mainly driven by time (Figure 1). In the fractionation method, the analytes of interest all elute within the first minute of the analysis. This causes very narrow peak widths and severe coelution. As a result, there will be little time for fragmentation and a high chance of complex MS/MS spectra. In contrast, the hydrophilic interaction liquid chromatography (HILIC) method demonstrates much more separation and also considerably broader peaks. Figure 1 demonstrates that there is even a slight separation within the structural isomer pairs using a HILIC separation. This means that in the HILIC method, there is more time for fragmentation and a lower chance of complex MS/MS spectra. The comparison of the two methods should demonstrate the influence of chromatographic separation and peak width on the selectivity of the DIA MS/MS spectra.

During the SWATH method development, we aimed for the highest selectivity by including as many windows as possible while still having sufficient data points across the chromatographic peak for absolute quantitation. We aimed for a minimum of ten data points, since this number is generally accepted to accurately describe a chromatographic peak [28]. For this, we calculated the maximum number of SWATH windows that still ensured ten data points on the narrowest chromatographic peak [16]. This resulted in a maximum of seven windows in the fractionation method and 30 windows in the HILIC method. The fixed SWATH methods were constructed by dividing the mass range by the number of SWATH windows. A one Dalton overlap between the windows was included to ensure the inclusion of all precursors in the desired mass range. The fixed SWATH window size was 25 (+1) and six (+1) Dalton in the fractionation and HILIC method, respectively. The window sizes of the variable SWATH methods were calculated using the density of all precursor ions in the desired mass range (75–250 *m*/*z*) [29]. The window sizes were adjusted in order to have an equal density of precursor ions in each SWATH window.

### 2.2. Quantitation of Structural Isomers

**Figure 2:**
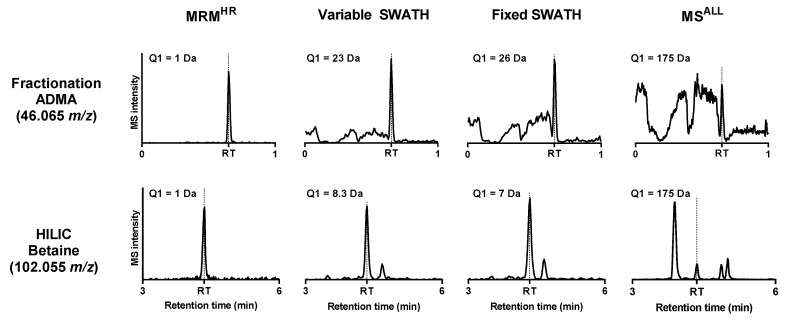
Extracted ion chromatograms of the different types of MS/MS scans with corresponding Q1 window sizes measured in positive ionization mode. The extraction width is 0.02 *m*/*z*. Fluctuating baselines can be observed in the fractionation method with increasing Q1 window sizes. Multiple peaks can be seen in HILIC with increasing Q1 window sizes.

Five structural isomer pairs were quantified by analyzing diagnostic product ions. DIA quantification accuracies were reported as a percentage of the MRM^HR^ quantification (see Formula (1) in Section 4.5). The accuracies of the best performing product ions are depicted in Table 2 and the performance of all product ions is presented in the supplementary information (Table S6). In general, more diagnostic product ions could be integrated reliably by SWATH in comparison with MS^ALL^. The extracted ion chromatograms in MS^ALL^ were often disturbed by fluctuating or high baselines, which led in some cases to the absence of certain product ion peaks. In general, the baseline signal increased with increasing Q1 windows (as shown by Figure 2). The elevated baselines indicated that background ions produced similar products as some of the structural isomers. Besides, extracted ion chromatograms of MS^ALL^ often showed multiple peaks, indicating the presence of common product ions produced by different precursors. In some cases, coelution of these common product ions obstructed proper peak integration. Similar integration problems occurred in the SWATH analysis. However, the frequency and severity of these problems were noticeably less. MRM^HR^ resulted in the cleanest chromatograms with low baselines and no overlapping peaks. These findings are in accordance with the work of Venable et al. in which it was shown that the appearance of background peaks and high background noise decreases with decreasing Q1 window sizes [30].

In MS^ALL^, six of the ten compounds had a quantification accuracy above 115% (124–1944%), indicating that multiple precursor ions indeed produced similar product ions. Especially the fractionation method was compromised by this particular DIA mode. The quantification values were considerably higher than MRM^HR^, which was expected because of the high degree of coelution and large window sizes. The analysis of complex matrices, in particular, is prone to suffer from product ion overlap because of the high chance of coelution of compounds with similar fragmentation patterns [29]. Although the product ions were diagnostic within an isomer pair, they lacked selectivity in the presence of a biological matrix. By decreasing the size of the Q1 windows in the SWATH analysis, the accuracies drastically improved. This applied to both the fractionation and HILIC method.

**Figure 3:**
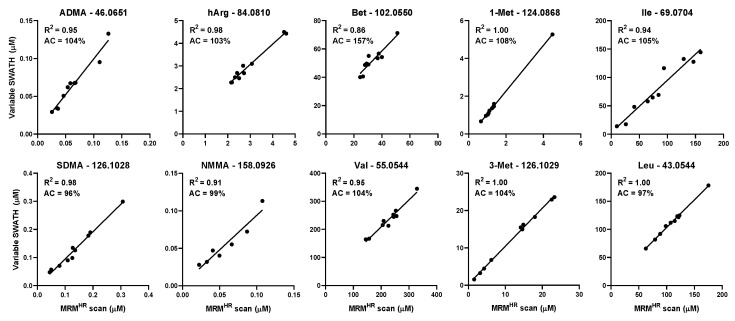
Correlation (R^2^) and accuracy (AC) of the quantification values of 10 structural isomers in 10 volunteers. Each data point represents the analyte concentration quantified by MRM^HR^ and variable SWATH. The quantification values of the best performing product ion are depicted in the graph. NMMA was not detected in three volunteers.

The variable SWATH method improved the quantification accuracies more than the fixed SWATH method in both chromatography methods. This demonstrates that even with a conventional LC separation and relatively small SWATH windows (6 + 1 Dalton), the selectivity is still dependent on the customization of the SWATH windows (fixed versus variable). The power of customized SWATH windows is emphasized by a comparison of the three MS/MS methods. Table 2 shows that 85, 60 and 30% of the individual isomer quantifications were good (>85% and <115%) in variable SWATH, fixed SWATH and MS^ALL^, respectively, indicating that a higher degree of Q1 window customization results in a more accurate quantification. The combination of variable SWATH and HILIC resulted in the accurate quantification of all investigated isomers. The quantitative performance of the latter platform was maintained when the five isomers pairs were quantified in blood samples of ten male donors (see Figure 3). The high correlation with MRM^HR^ demonstrated that differences in biological samples did not interfere with the selectivity of the quantification of the selected isomers. Figure 3 shows that the correlation coefficient of nine structural isomers was higher than 0.90, with a mean quantification accuracy of 102% (96–108%) per diagnostic fragment. The strongly correlated and highly accurate quantification values demonstrate that the quantification accuracies of variable SWATH and HILIC are not affected by a large variation in blood sample matrices. Figure S2 and Table S7 in the Supplementary Information reveal that the other diagnostic product ions followed the same trend. The only product ion that behaved differently was the product ion of betaine. Even though the quantification values were strongly correlated between MRM^HR^ and variable SWATH (*R*^2^ = 0.86), the mean quantification accuracy was 157%. Since the relative standard deviation of the quantification accuracies throughout the ten blood samples was low (9%) and the correlation with MRM^HR^ high, it was expected that the quantification values were structurally increased in all volunteers rather than influenced by product ions derived from the blood samples. However, the exact mechanism of this increase remained unclear.

In accordance with our results, Zhang et al. demonstrated a performance improvement by using variable SWATH windows over fixed SWATH windows. The performance improvement was found in both the identification and quantification of peptides [12]. Table 2 also demonstrates that DIA methodologies benefit from extensive chromatographic separations, since DIA fragmentation in the HILIC method resulted in a more accurate quantification. Especially MS^ALL^ analyses require a thorough separation, because the size of the Q1 window adds little extra selectivity [13]. In addition to DIA type and chromatographic separation, MS/MS quantifications improve with increasing peak widths because smaller SWATH windows can be applied resulting in a more selective fragmentation [12]. This has to be taken into account when DIA protocols are implemented in high-throughput platforms in which severe coelution and small peak widths often occur. Our results show that using a HILIC separation variable SWATH, nine out of 10 structural isomers can be quantitated accurately in a robust manner. However, it does not mean that all product ions in this methodology are selective. Table S6 in the supplementary information shows that there are still eight out of the 28 product ions that could not be accurately quantified. Therefore, the possibility of product ion overlap should always be considered when DIA methodologies are used for quantification.

### 2.3. Identification of Metabolites

In metabolomics, untargeted analyses can be performed to correlate unknown features with a certain physiological effect. Once a feature demonstrates a correlation with an investigated response, it is important to unravel its structure in order to understand its biochemical mechanism in the studied effect. Identification strategies are crucial to transform data (*m*/*z* values) into information (chemical structures). MS/MS data of unknown features can be used to search for potential candidates in commercially available spectral libraries, like NIST. Here, we discuss the qualitative power of DIA-acquired MS/MS spectra using a spectral library search.

The library hit scores and number of correct library hits using SWATH and MS^ALL^ are listed in Table 3. The identities of the metabolites were known in advance, which facilitated the evaluation of the correctness of the structure annotation. As shown by Table 3, practically all identifications were correct using a HILIC separation and SWATH. This confirms the qualitative power of SWATH, which has been shown numerous times before [31,32,33]. The identification in our HILIC method and the latter referenced literature all make use of a conventional chromatographic separation, which results in relatively clean MS/MS spectra. In contrast, the fractionation method, which makes use of a limited separation, demonstrated a decrease in qualitative performance as five and six of the 20 compounds could not be identified in variable and fixed SWATH, respectively. The higher degree of coelution and large window sizes resulted in more complex MS/MS spectra. Deconvolution was applied to remove several impurities from the MS/MS scan, as it is known that this decreases the complexity of the DIA-based MS/MS scans [34]. However, deconvolution has a limited power when severe coelution and wide Q1 windows dissociate the link between the precursor and product ion. A separation based on fractions results often in identical retention times for several precursors, which makes it difficult to link a precursor ion to its product ions by retention time.

**Figure 4:**
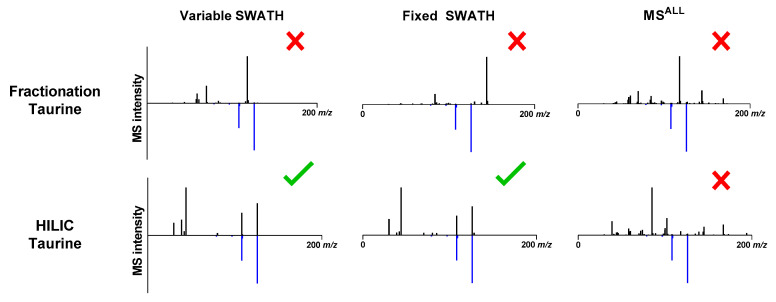
Experimental (black) and NIST library (blue) MS/MS spectra comparison measured in positive ionization mode. The HILIC separation in combination with SWATH resulted in the assignment of the correct structure.

As shown in Figure 4, MS/MS spectra become more complex with increasing Q1 window sizes, which is clearly demonstrated by a comparison of the SWATH and MS^ALL^ spectra. This explains the fact that MS^ALL^ only allowed for the unequivocal identification of nine and 13 of the 20 compounds in the fractionation and HILIC method, respectively. The identification of taurine in Figure 4 illustrates the problems of coelution and large window sizes because only a HILIC separation using SWATH resulted in a correct identification. Although the two most abundant MS/MS peaks of the library spectra were present in all DIA-acquired spectra, these peaks were overshadowed by numerous other higher intensity peaks. This obstructed the correct identification of taurine.

The qualitative power of SWATH clearly outperformed MS^ALL^, which has been shown before [35]. The identification performance did not seem to be affected by a higher degree of SWATH window customization as the number of correct hits and library hit scores were virtually similar between variable and fixed SWATH. This is in contrast to a study of Zhang et al., in which variable Q1 windows improved the selectivity of the SWATH analysis of metabolites in urine [12]. The investigated mass range in the latter study, however, was substantially higher than in our study (50–915 *m*/*z* versus 75–250 *m*/*z*). As a result, the difference in ion density throughout the mass range was considerably higher resulting in a larger variation in variable window sizes in order to equalize the ions density in each SWATH windows. Therefore, the fixed and variable SWATH window size differed to greater extent than in our study. This resulted in a more prominent difference in the quality and identification efficiency of the acquired SWATH spectra.

## 3. Conclusions

Data-independent acquisition (DIA) is an attractive technique in mass spectrometry-based metabolomics. It offers the user the possibility to acquire fragmentation data of all analytes in a defined mass range without introducing a bias towards certain analytes. However, the possibility of product ion overlap and complex MS/MS spectra may impair the quantitative and qualitative performance of these MS/MS scans. Our work demonstrates that the performance of DIA methodologies is highly dependent on the type of chromatography and the organization of the Q1 filters. Fast chromatography in combination with wide Q1 filters tends to result in fragmentation data that are often unusable for qualitative and quantitative purposes. In contrast, smaller-sized Q1 windows (i.e., SWATH) in combination with hydrophilic interaction liquid chromatography (HILIC) separation resulted in correct metabolite identifications and more often in accurate quantification values. Especially customized SWATH windows (i.e., variable SWATH) demonstrated a superior performance, since it allowed for the accurate quantification of 10 structural isomers. Apart from one compound, the quantification accuracies remained good when these isomers were quantified in ten highly diverse blood samples demonstrating the robustness of the analysis. Although a window size of 25 Dalton is common practice in proteomics, we have experienced inaccurate quantifications using fixed SWATH windows of six Daltons in our metabolomics application. Therefore, it seems that the density of precursor ions per mass unit is higher in metabolomics compared to proteomics. Since we have shown good qualitative and quantitative performances using variable SWATH windows of around six Dalton, we would recommend the use of this window size in metabolomics applications in order to maintain a proper selectivity and speed during fragmentation. The use of variable SWATH windows and a HILIC separation are promising for combined targeted and untargeted platforms, since it allows for both quantification and identification. Targeted platforms involving SWATH can be re-interrogated at a later stage to identify interesting unknown features. On the other hand, untargeted platforms using SWATH can also be used to quantify critical isomer pairs. The combination of targeted and untargeted metabolomics platforms should facilitate the analytical method development workflow and increase the information provided by a single analysis.

## 4. Materials and Methods

### 4.1. Chemicals

Standards were purchased from HMDB (Edmonton, AB, Canada), Sigma-Aldrich (Zwijndrecht, The Netherlands) and Fluka (Seelze, Germany). Internal standards were purchased from Cambridge Isotopes (Tewksbury, MA, USA), Cortecnet (Voisins-Le-Bretonneux, France) and CDN Isotopes (Nieuwegein, The Netherlands). An overview of the (internal) standards and concentrations is provided in the supplementary information (Tables S1 and S2).

Acetonitrile (LC-MS grade) was purchased from Biosolve B.V. (Valkenswaard, The Netherlands) and methanol (Ultra-LC-MS grade) was purchased from Actu-All (Oss, The Netherlands). Ammonium formate (≥99.995%) and ammonium acetate (≥99.0%) were purchased from Sigma-Aldrich (Zwijndrecht, The Netherlands). Ammonium hydroxide (28–30 wt.% solution of ammonia in water) and formic acid (98%) were purchased from Acros Organics (Bleiswijk, The Netherlands). Pooled heparin plasma (June 2018) was used for quantitative and qualitative performance evaluation and purchased from Sanquin (Amsterdam, The Netherlands). Ten diverse EDTA plasma samples were purchased from Bio IVT (Westbury, NY, USA). The clinical variables are mentioned in the supplementary information (Table S3).

### 4.2. Standard and Internal Standards

Individual (internal) standards were dissolved in water. Calibration and internal standard solutions were prepared by mixing the individual standards into a final solvent composition of 75% methanol in water. The C8 calibration solution was the highest concentration and the subsequent calibration solutions (C7–C1) were 1:1 dilutions of the previous solution. The C0 calibration solution was a 75% methanol solution that did not contain the calibration standards. The internal standard and C4 calibration concentration were set to mimic the physiological concentration as found in HMDB [36]. The internal standard choice for the correction of the isomers was based on achieving the highest linearity and precision for the corresponding isomer.

### 4.3. Sample Preparation

During sample preparation, 15 µL of heparin plasma, 15 µL of internal standard solution and 15 µL of calibration standard solution were mixed with 30 µL of methanol by vortex mixing. Non-spiked samples were prepared by replacing the calibration standard solution by 15 µL of 75% methanol. Subsequently, the samples were centrifuged at 4 °C and 16,100× *g* for 10 min. Fifty microliters of supernatant was transferred into a 1.5 mL HPLC vial containing a 150 µL insert.

### 4.4. LC-MS

A Sciex X500R QToF (Darmstadt, Germany) was used for the MS analysis (resolution ranging from 30 K to 40 K [37]). A Shimadzu Nexera UHPLC (Darmstadt, Germany) was extended with an Agilent 1260 infinity isocratic pump (Waldbronn, Germany) and two VICI six-port valves (Rotterdam, The Netherlands).

The high-throughput analyses were performed by an on-line fractionation method, which has been published before [27]. In short, a ZORBAX Extend-C18 2.1 × 5 mm, Sepax SCX 2.1 × 10 mm and Sepax WAX 2.1 × 10 mm were serially coupled in order to trap apolar, positively and negatively charged analytes, respectively. Three mobile phases are used for loading (0.2% formic acid in water), ion-exchange elution (100 mM ammonium acetate pH 10 in water) and C18 elution (2 mM ammonium acetate in methanol). The flow rate was 800 µL/min for loading/C18 elution and 500 µL/min for ion-exchange elution. The injection volume was set at 1 µL. The polarity, ion source gas 1, ion source gas 2, curtain gas, CAD gas, temperature, declustering potential and spray voltage were set at positive, 40 psi, 60 psi, 40 psi, 7 psi, 650 °C, 80 V and 5500 Volt, respectively.

The conventional LC analysis was performed by a HILIC separation. The HPLC column was a SeQuant ZIC-HILIC 2.1 × 100 mm column (Amsterdam, The Netherlands). Mobile phase A consisted of 10 mM ammonium formate and 0.075/90/10 (*v*/*v*/*v*) formic acid/acetonitrile/water, and mobile phase B consisted of 10 mM ammonium formate and 0.075/10/90 (*v*/*v*/*v*) formic acid/acetonitrile/water. The gradient started at 0% B. After 1.2 min the gradient linearly increased to 75% B in 7.96 min. The gradient was kept at 75% B for 4.84 min in order to flush the column. Subsequently, the gradient decreased to 0% B in 0.2 min and was kept at this value for 3.8 min to equilibrate the column. The flow rate and the injection volume were set at 500 µL/min and 3 µL, respectively. The polarity, ion source gas 1, ion source gas 2, curtain gas, CAD gas, temperature, declustering potential and spray voltage were set at positive, 40 psi, 60 psi, 35 psi, 7 psi, 575 °C, 80 V and 5500 Volt, respectively.

All MS methods consisted of a full scan of 50 ms at a collision energy 5 eV followed by at least one MS/MS scan of 30 ms at a collision energy of 20 eV. The MRM^HR^ analysis was conducted in product ion scan mode using Sciex OS 1.5 (Darmstadt, Germany). MS^ALL^ was performed in SWATH mode using one MS/MS window ranging from *m*/*z* 75 to 250. The SWATH analysis in the fractionation and HILIC method consisted of seven and 30 SWATH windows, respectively. Variable SWATH windows were calculated using the “SWATH Variable Window Calculator V1.1” from Sciex (Supplementary Information Figure S1). The windows sizes of the variable and fixed SWATH methods are depicted in the supplementary information (Tables S4 and S5). The cycle time of the MRM^HR^, SWATH and MS^ALL^ methods was 0.272, 0.339 and 0.140 s, respectively.

### 4.5. Diagnostic Product Ions and Quantification of Structural Isomers

Structural isomer standards in water were analyzed individually by a flow injection analysis and an MRM^HR^ scan in order to find the corresponding product ions (see Figure S3 in the Supplementary Information). Product ions and corresponding intensities were obtained by taking the average mass spectrum of the analyte peak. All product ions that demonstrated an intensity of at least 1 percent of the most abundant product ion were included. Overlapping product ions (similar exact mass) within an isomer pair were excluded. The remaining diagnostic product ions were used for the quantification of the structural isomers.

Diagnostic product ions were quantified by the fractionation and HILIC method by plotting the peak area ratio of a diagnostic product ion against the concentration. Nine different calibration points were used to construct the calibration curve (C0–C8). All calibration points were used for the calibration curve, unless they were outside of the linear range (R^2^ < 0.99). Five replicates of a plasma pool were analyzed to determine the physiological concentrations. Product ions that were included for quantification demonstrated no peak overlap with other common product ions, had a linear calibration curve (≥0.99) and deviated less than 15% (*n* = 5). Each analyte was quantified using four different MS methods: MRM^HR^, variable SWATH, fixed SWATH and MS^ALL^. The MRM^HR^ scan was taken as the reference value. Quantification accuracies are reported as a percentage of the MRM^HR^ scan (see Equation (1)).

(1) $$Quantification\;accuracy=\;\frac{\left[DIA\;protocol\right]}{\left[MRM^{HR}\right]}\times 100\%$$

The remaining diagnostic product ions demonstrated a quantification accuracy of 85–115%. The robustness of the HILIC and variable SWATH method was assessed by determining the quantification accuracies of the remaining diagnostic product ions in blood samples of ten male donors. The subjects were selected based on their differences in clinical variables, i.e., age, race, BMI, fasting state and smoking habit (see Supplementary Information Table S3). The diversity in the blood samples should push the performance of the HILIC and variable SWATH method to its extremes.

### 4.6. Identification of Metabolites

The NIST 2017 library was used to evaluate the qualitative power of the DIA MS/MS spectra. The library was uploaded in Library View and accessed via Analytics in Sciex OS. Deconvolution was applied by Analytics to remove product ions that did not follow the same intensity trend as the precursor ion. The removal of these product ions resulted in a decreased MS/MS spectra complexity and an improved recovery of the relative product ion intensities. The precursor mass tolerance was set at 0.02 Dalton. The identification was performed by a candidate search, which shows the best spectral match in the selected library with a corresponding library score. The best spectral match and the library score were obtained using the square roots of the mass-weighted intensities of the unknown masses (*U_m_*) and the library masses (*L_m_*) (see Equation (2)) [38].

(2) $$Library\;score=\frac{{\left(\sum^{\;}U_{m}\times L_{m}\right)}^{2}}{\sum^{\;}U_{m}^{\;2}\times \sum^{\;}L_{m}^{\;2}}\;\times 100\%$$

This formula is based on the dot-product (cosine) algorithm, which is widely applied in MS/MS spectral matching [39]. In Analytics, the “fit” function was applied to Formula (2). This function only allows the inclusion of masses that are present in the library spectrum. The corresponding library score is a measure of the degree to which the library spectrum is contained in the unknown spectrum. The “fit” function tends to give higher scores to complex MS/MS spectra in comparison with the “purity” function, which includes all unknown masses. DIA spectra are often considered as complex due to the presence of product ions originating from other precursor ions. Therefore, the “fit” function is highly suitable to score library hits resulting from DIA spectra. Twenty known metabolites were identified using five technical replicates. The correct identity and retention time were found by evaluating the MRM^HR^ data. The mean library hit score and the number of correct hits were noted. The absence of a library match and an incorrect structural assignment were given a library hit score of 0%.

## Figures and Tables

**Table 1:** Overview of the most commonly used tandem mass spectrometry modes. MRM: multiple reaction monitoring, MRM^HR^: high-resolution multiple reaction monitoring, PRM: parallel reaction monitoring, DDA: data-dependent acquisition, DIA: data-independent acquisition, SWATH: sequential window acquisition of all theoretical mass spectra and AIF: all-ion fragmentation. Q1 (first quadrupole) size, resolution, precursor selection and targeted/untargeted profile are indicated.

Name	MRM	MRM^HR^/PRM	DDA	DIA	
				SWATH	MS^ALL^/MS^E^/AIF
Q1 size	Narrow	Narrow	Narrow	Medium	Wide
High/low resolution	Low	High	High	High	High
Precursor selection	User-defined	User-defined	Based on parent ion intensity *	No selection	No selection
Targeted/untargeted	Targeted	Targeted	Untargeted	Untargeted	Untargeted

* Can also be based on, e.g., mass defect or isotope pattern.

**Table 2:** The quantification accuracy of structural isomers in comparison with MRM^HR^. Compounds that could not be quantified due to an insufficient linearity (<0.99), high variability (>15%) or integrations problems (peak overlap or too high baseline) are indicated by the zero values.

Analyte	Variable SWATH		Fixed SWATH (%)		MS^ALL^ (%)	
	Fractionation	HILIC	Fractionation	HILIC	Fractionation	HILIC
ADMA	88	96	0	102	0	0
SDMA	107	101	96	110	106	0
hArg	109	99	389	102	271	0
NMMA	739	111	6383	226	1944	0
Bet	106	102	103	134	136	124
Val	98	91	93	91	99	101
1-Met	157	113	0	109	0	102
3-Met	97	99	97	89	193	89
Ile	106	112	113	99	129	90
Leu	144	110	148	117	171	168

Accuracies were divided into three groups: 85–115% (green), 115–120% (yellow) and >120% (red).

**Table 3:** Identification of twenty known metabolites using SWATH and MS^ALL^. The experimental MS/MS spectra were compared with the NIST 2017 library. The mean score of the library hits (*n* = 5) and the number of correct hits are listed in the table. MS/MS spectra that could not be matched or resulted in a wrong hit were given a score of 0%.

Analyte	Variable SWATH				Fixed SWATH				MS^ALL^			
	Frac		HILIC		Frac		HILIC		Frac		HILIC	
	Score (%)	Hits (#)	Score (%)	Score (%)	Score (%)	Hits (#)	Score (%)	Hits (#)	Score (%)	Hits (#)	Score (%)	Hits (#)
Proline	100	5/5	100	5/5	100	5/5	100	5/5	100	5/5	100	5/5
Arginine	100	5/5	100	5/5	0	0/5	99	5/5	100	5/5	99	5/5
Acetyl-carnitine	99	5/5	99	5/5	98	5/5	99	5/5	96	5/5	98	5/5
Creatinine	100	5/5	99	5/5	100	5/5	99	5/5	95	5/5	99	5/5
Ornithine	0	0/5	99	5/5	0	0/5	99	5/5	40	2/5	96	5/5
Methionine	76	5/5	99	5/5	0	0/5	99	5/5	0	0/5	99	5/5
Carnitine	98	5/5	98	5/5	96	5/5	98	5/5	78	5/5	97	5/5
Citrulline	98	5/5	97	5/5	99	5/5	94	5/5	0	0/5	50	3/5
Tyrosine	94	5/5	97	5/5	94	5/5	96	5/5	92	5/5	98	5/5
Phenylalanine	76	5/5	96	5/5	57	4/5	96	5/5	41	3/5	97	5/5
Histidine	98	5/5	95	5/5	98	5/5	95	5/5	99	5/5	3	1/5
Serine	0	0/5	93	5/5	0	0/5	97	5/5	0	0/5	19	3/5
Tryptophan	63	5/5	93	5/5	77	5/5	91	5/5	0	0/5	94	5/5
Glutamine	93	5/5	93	5/5	92	5/5	94	5/5	97	5/5	93	5/5
Cystine	88	5/5	92	5/5	92	5/5	91	5/5	0	0/5	93	5/5
Taurine	0	0/5	92	5/5	0	0/5	89	5/5	0	0/5	0	0/5
Lysine	0	0/5	91	5/5	88	5/5	89	5/5	0	0/5	93	5/5
Asparagine	0	0/5	82	5/5	0	0/5	92	5/5	0	0/5	0	0/5
Glutamic acid	90	5/5	62	4/5	94	5/5	82	5/5	97	5/5	0	0/5
γ-butyrobetaine	54	5/5	44	5/5	73	5/5	60	5/5	0	0/5	0	0/5
Average	66		91		63		93		47		66	

# indicates the number of correct hits. The colors also indicate the number of correct hits, ranging from dark green (5/5 correct hits) to red (0/5 correct hits).

## Supplementary Materials

The following are available online at https://www.mdpi.com/2218-1989/10/12/514/s1, Figure S1: Variable window calculator results, Figure S2: The quantification values of 10 structural isomers in 10 volunteers, Figure S3: Product ions of the 10 structural isomers measured by a flow injection analysis and MRMHR, Table S1: Standards and internal standard correction, Table S2: Internal standards, Table S3: Clinical variables of ten healthy male subjects, Table S4: SWATH window sizes for the fractionation method, Table S5: SWATH windows sizes for the HILIC method, Table S6: The quantification accuracy of structural isomers, Table S7: Correlation and accuracy of the quantification values of structural isomers in 10 volunteers.
