# Supplementary material for: Data-Independent Acquisition for the Quantification and Identification of Metabolites in Plasma
Source: Metabolites. 2020 Dec 18;10(12):514. doi: 10.3390/metabo10120514 (PMC7766927; doi:10.3390/metabo10120514)
Supplement: Supplementary file 1 [file metabolites-10-00514-s001.pdf]

Data independent acquisition for the quantification and identification of metabolites in plasma

Tom van der Laan, Isabelle Boom, Joshua Maliepaard, Anne-Charlotte Dubbelman, Amy Harms and Thomas Hankemeier

* Correspondence: hankemeier@lacdr.leidenuniv.nl; Tel.: +31-71-527-4226/4220

Contents:

Figure S1: Variable window calculator results

Figure S2: The quantification values of 10 structural isomers in 10 volunteers

Figure S3: Product ions of the 10 structural isomers measured by a flow injection analysis and MRM^{HR}

Table S1: Standards and internal standard correction

Table S2: Internal standards

Table S3: Clinical variables of ten healthy male subjects

Table S4: SWATH window sizes for the fractionation method

Table S5: SWATH windows sizes for the HILIC method

Table S6: The quantification accuracy of structural isomers

Table S7: Correlation and accuracy of the quantification values of structural isomers in 10 volunteers

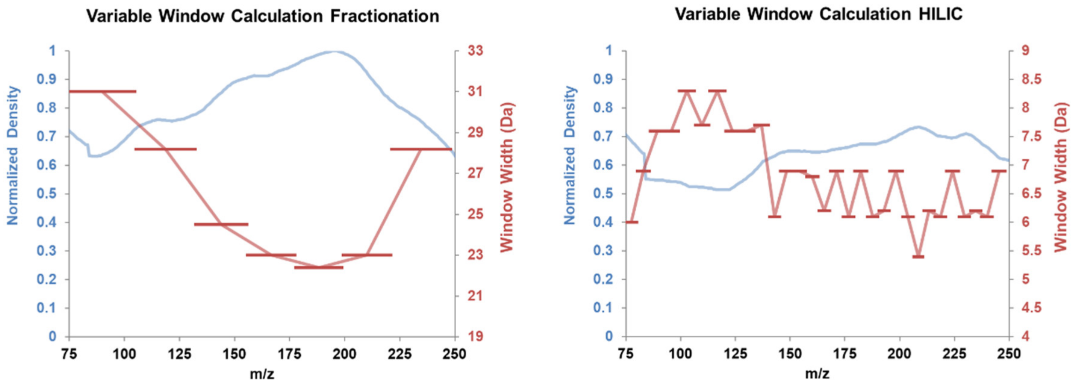

Figure S1. Variable window calculator V1.1 results. The left y-axis demonstrates the normalized density of the MS intensities of a full scan (75–250 *m*/*z*). The right y-axis demonstrates the window width of the calculated SWATH windows. The HILIC method has 30 windows and the fractionation method has 7 windows.

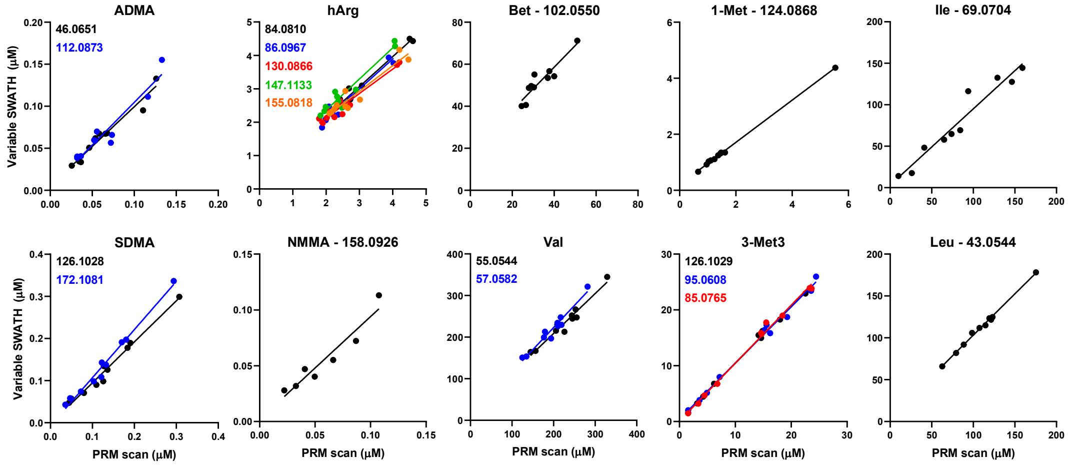

Figure S2. The quantification values of 10 structural isomers in 10 volunteers. Each data point represents the analyte concentration quantified by MRM^{HR} and variable SWATH. Different diagnostic product ions of one structural isomer are plotted in different colours.

Figure S3. Product ions of the 10 structural isomers measured by a flow injection analysis and MRM^{HR}. The injection volume was set at 1 μL. The flow rate and composition of the mobile phase was 800 μL/min and 0.1% formic acid in water, respectively.

Bet (Q1 = 118.1 *m/z*)

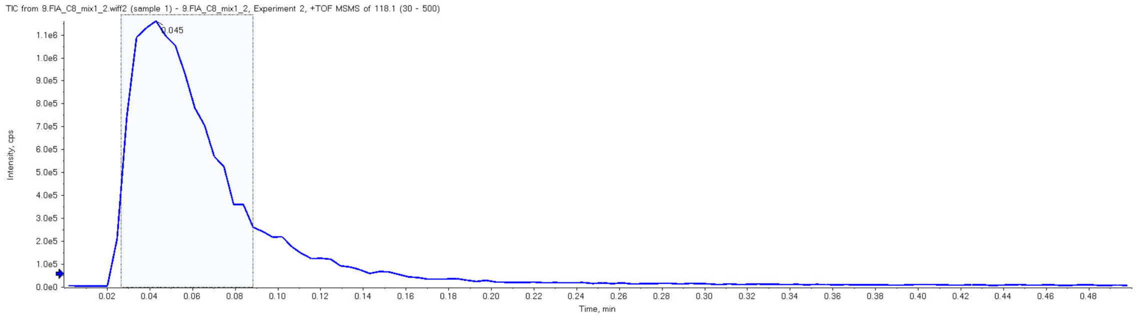

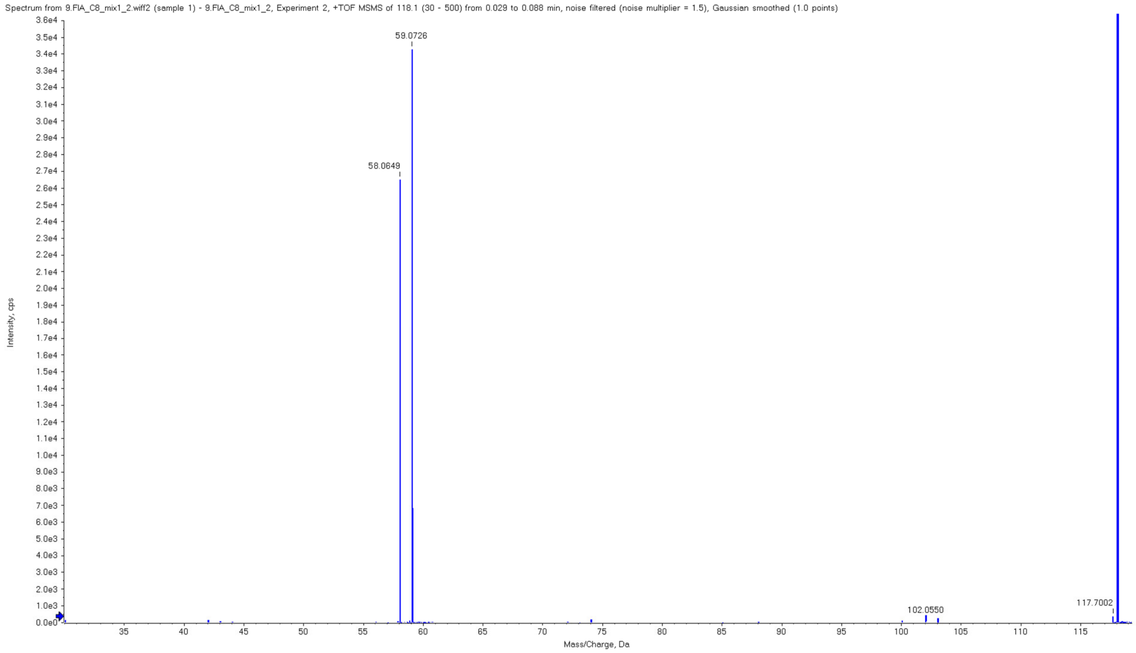

Mass/Charge (Da)	Height	Intensity Relative to Most Abundant Product Ion (%)
58.06492812	26491.17	77.30
59.07262482	34268.77	100.00
102.0550027	423.8501	1.24
117.700175	356.6517	1.04

Ile (Q1 = 132.1 *m*/*z*)

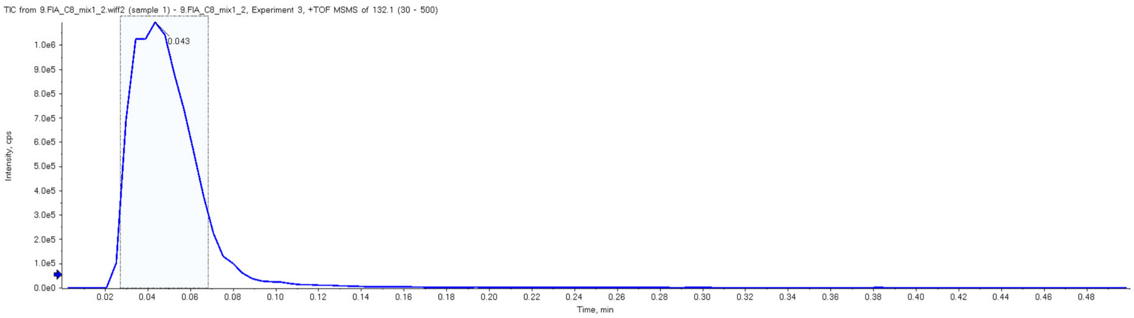

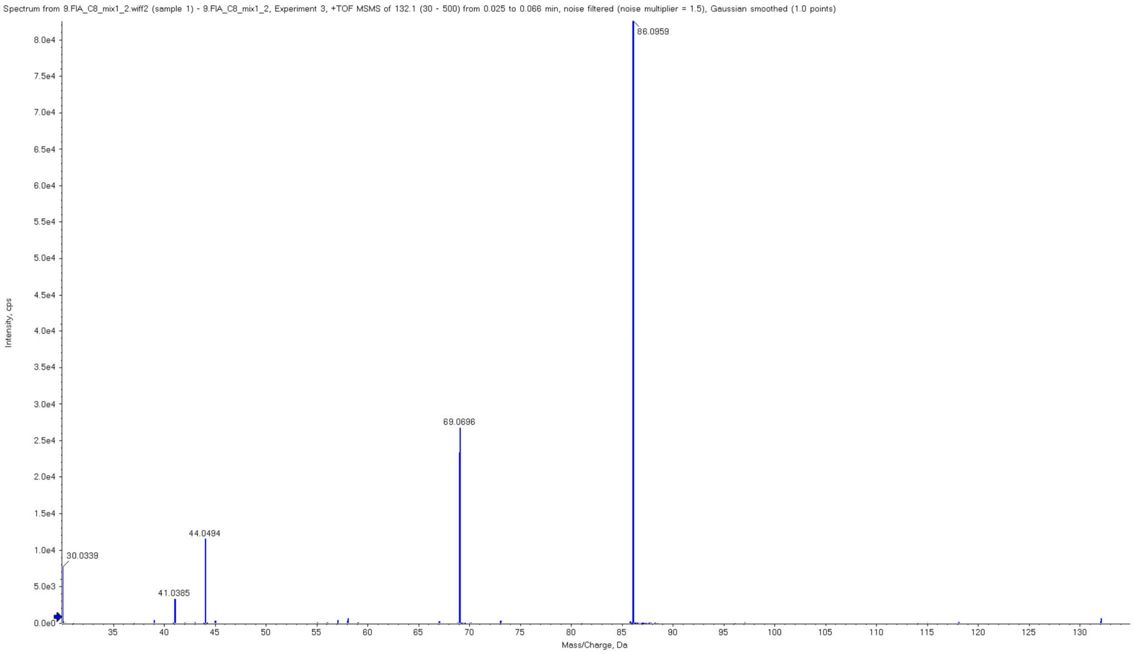

Mass/Charge (Da)	Height	Intensity Relative to Most Abundant Product Ion (%)
30.03385	7710.88	9.34
41.0385	3294.343	3.99
44.04941	11541.89	13.98
69.06965	26746.81	32.39
86.09588	82578.1	100.00

1-met (Q1 = 170.1 *m/z*)

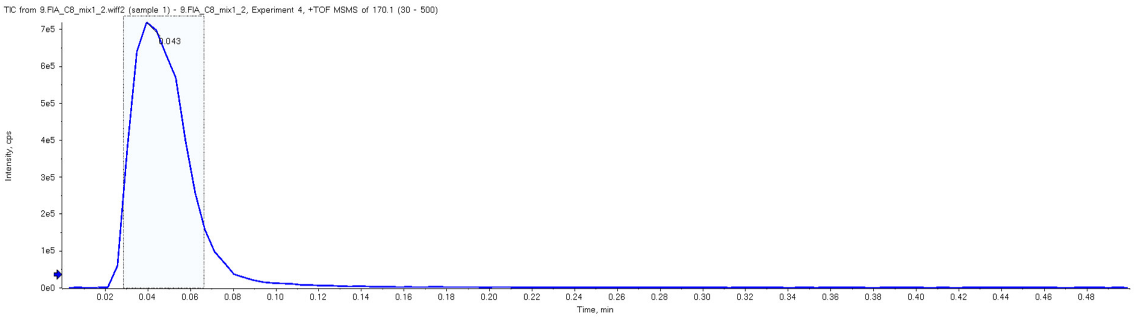

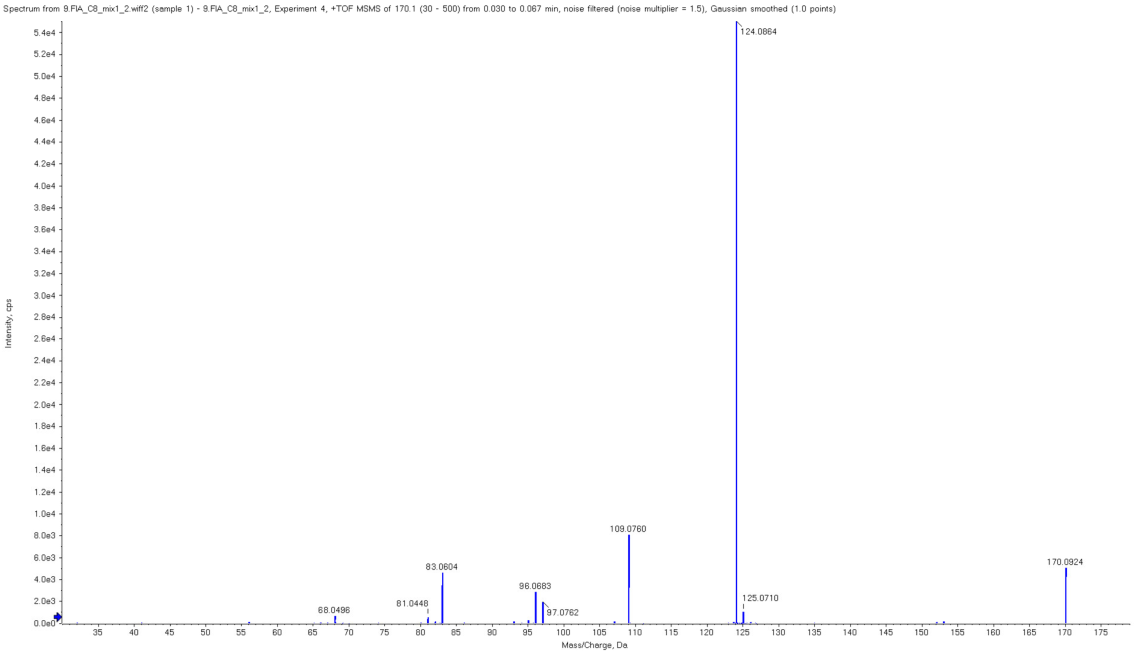

Mass/Charge (Da)	Height	Intensity Relative to Most Abundant Product Ion (%)
68.04961	667.2403	1.21
81.0448	584.0524	1.06
83.06039	4650.43	8.45
96.06831	2855.065	5.19
97.07618	1946.076	3.54
109.076	8118.871	14.75
124.0864	55046.24	100.00
125.071	1049.859	1.91
170.0924	5079.9	9.23

hArg (Q1 = 189.1 *m/z*)

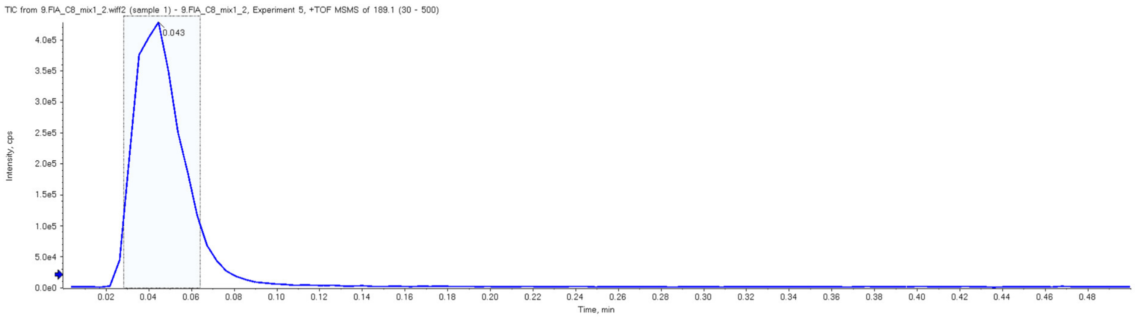

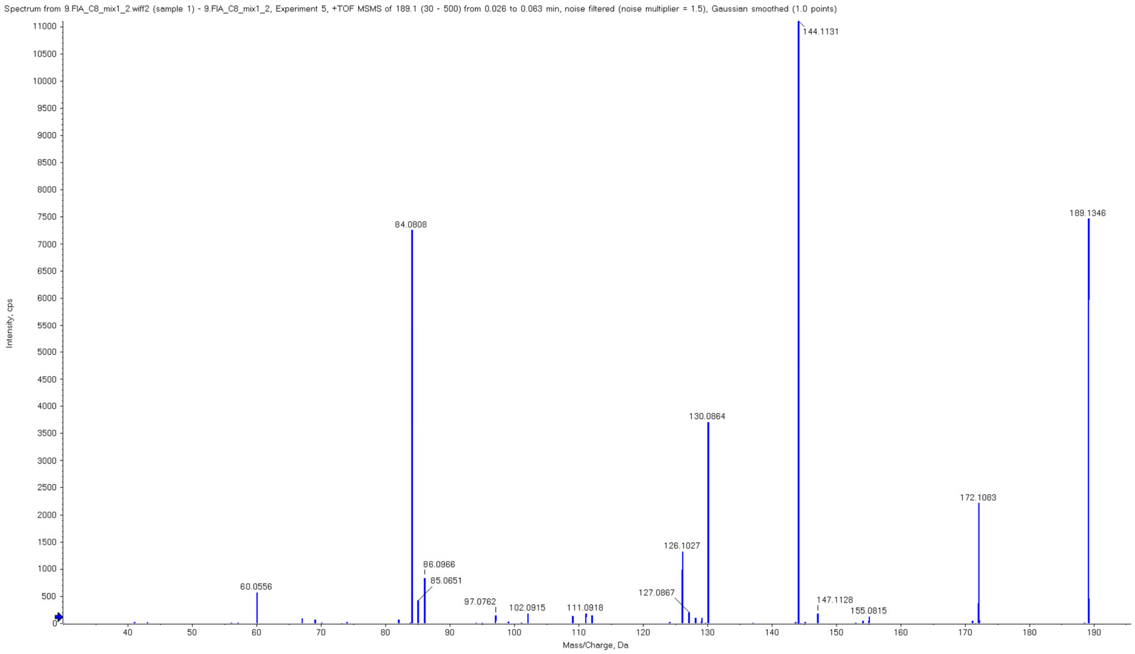

Mass/Charge (Da)	Height	Intensity Relative to Most Abundant Product Ion (%)
60.05563	565.748	5.09
84.08085	7257.251	65.35
85.06507	426.1838	3.84
86.09663	839.9134	7.56
97.07621	150.9142	1.36
102.0915	179.6448	1.62
109.076	136.3661	1.23
111.0918	177.9955	1.60
112.0757	143.8391	1.30
126.1027	1319.733	11.88
127.0867	207.0097	1.86
130.0864	3714.538	33.45
144.1131	11105.84	100.00
147.1128	184.7399	1.66
155.0815	129.1269	1.16
172.1083	2216.992	19.96

ADMA (Q1 = 203.2 *m/z*)

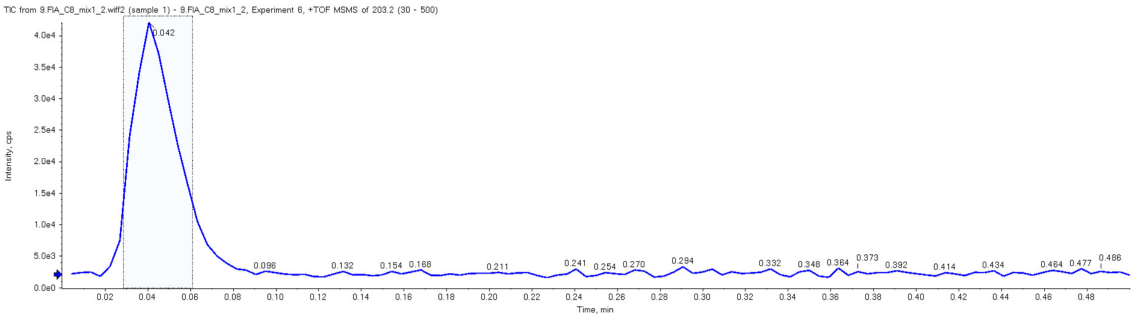

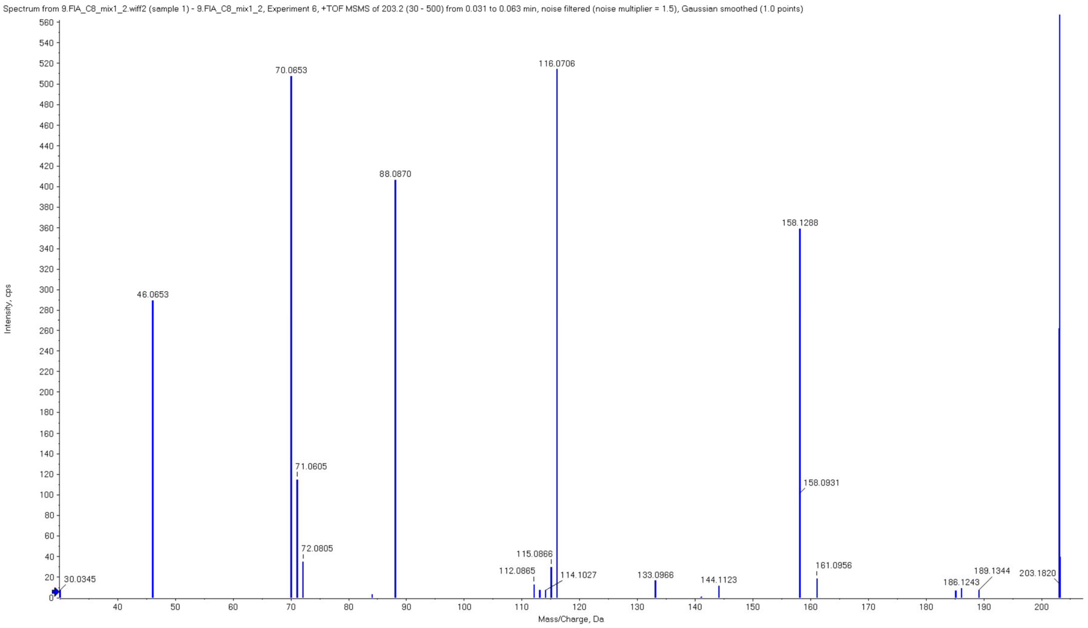

Mass/Charge (Da)	Height	Intensity Relative to Most Abundant Product Ion (%)
30.0345	7.654406	1.49
46.06529	289.8856	56.34
70.06529	507.7214	98.68
71.06055	115.0065	22.35
72.08045	35.08707	6.82
88.08703	406.9714	79.10
112.0865	13.23653	2.57
113.0704	7.715463	1.50
114.1027	7.464945	1.45
115.0866	29.99861	5.83
116.0706	514.5206	100.00
133.0966	16.94523	3.29
144.1123	11.80866	2.30
158.0931	101.4437	19.72
158.1288	359.3379	69.84
161.0956	18.62206	3.62
185.1385	7.238234	1.41
186.1243	9.598843	1.87
189.1344	7.745709	1.51

Leu (Q1 = 132.1 *m*/*z*)

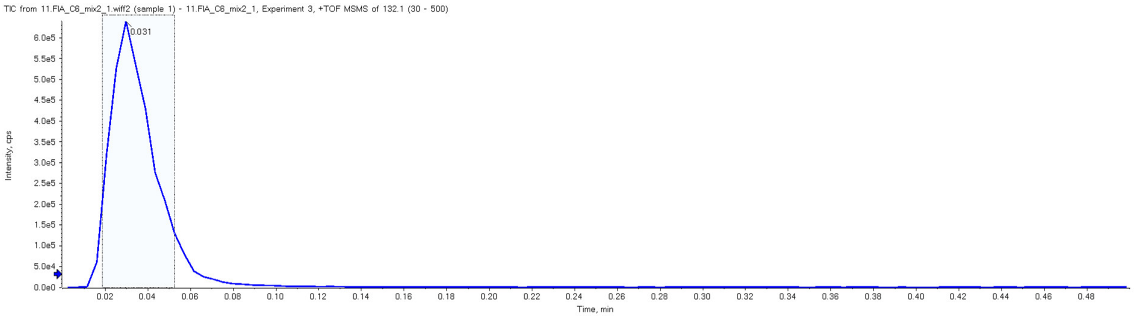

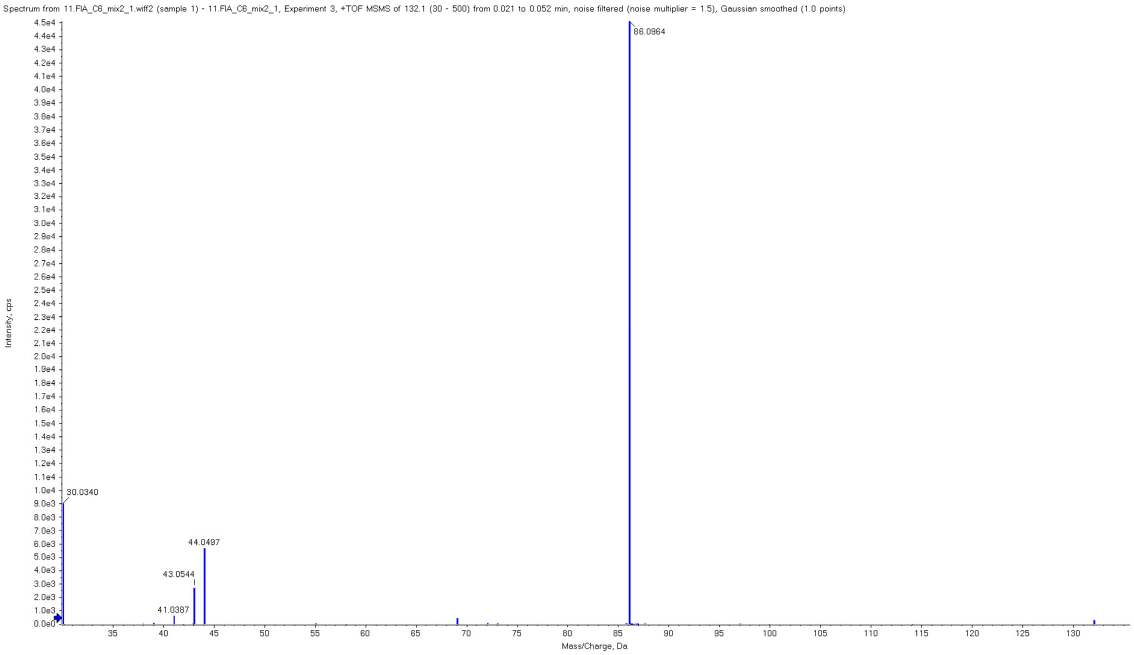

Mass/Charge (Da)	Height	Intensity Relative to Most Abundant Product Ion (%)
30.03397	9037.762	20.02
41.03875	629.4007	1.39
43.05445	2724.427	6.04
44.04967	5702.771	12.63
86.09637	45137.78	100.00

Val (Q1 = 118.1)

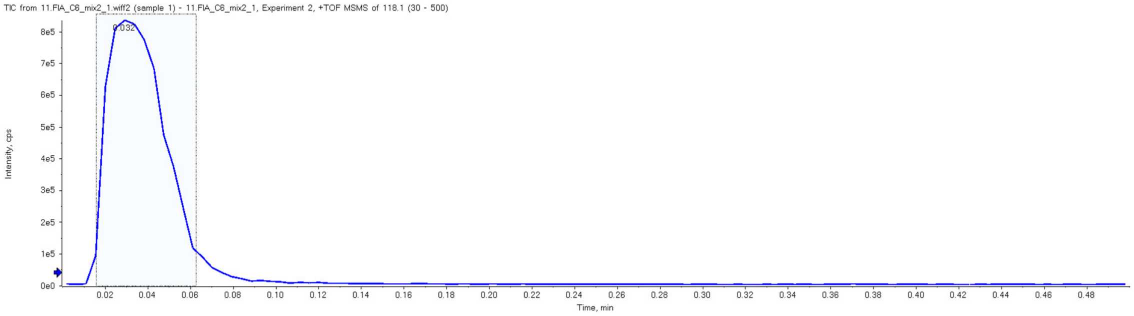

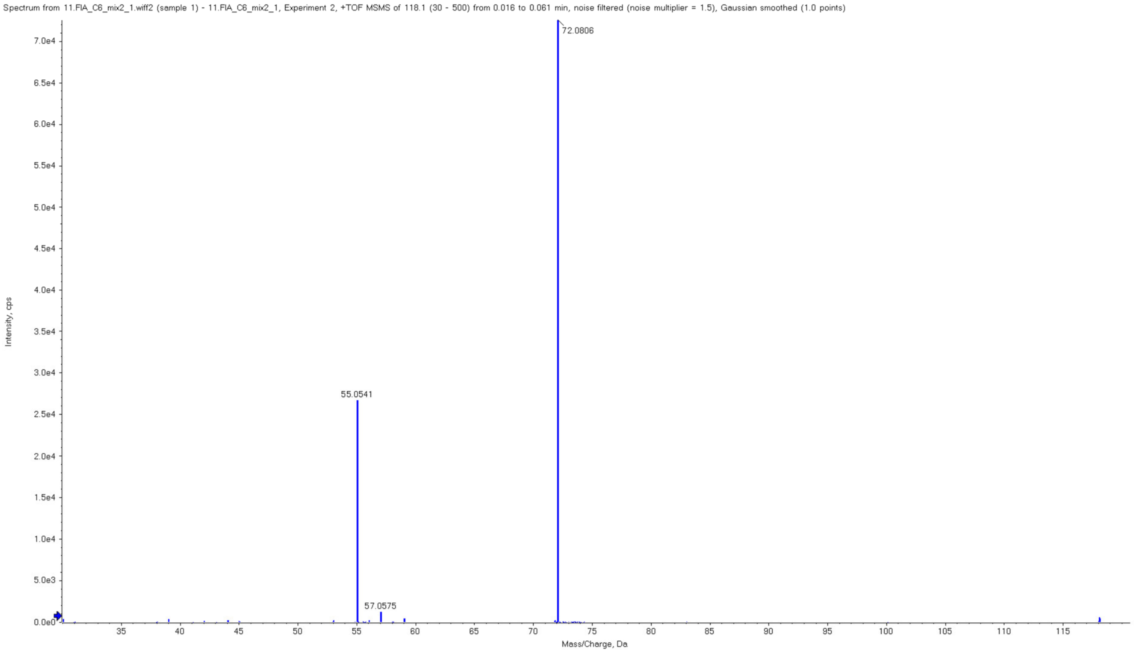

Mass/Charge (Da)	Height	Intensity Relative to Most Abundant Product Ion (%)
55.05410546	26690.9	36.78
57.0574615	1262.229	1.74
72.08055652	72569.6	100.00

3-met: (Q1 = 170.1)

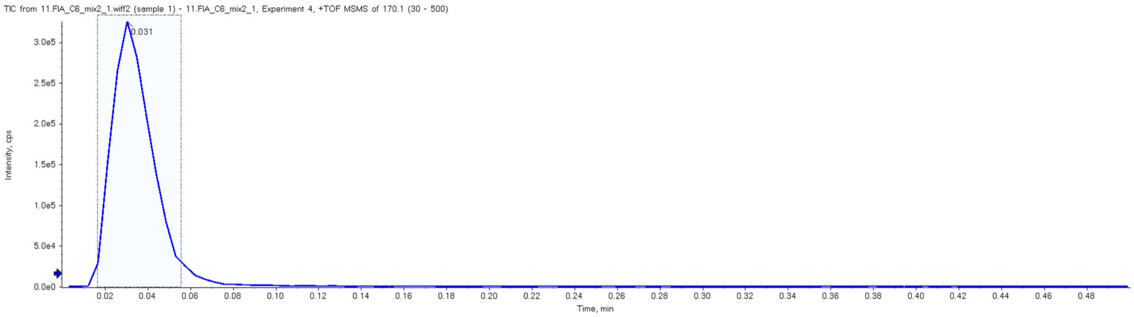

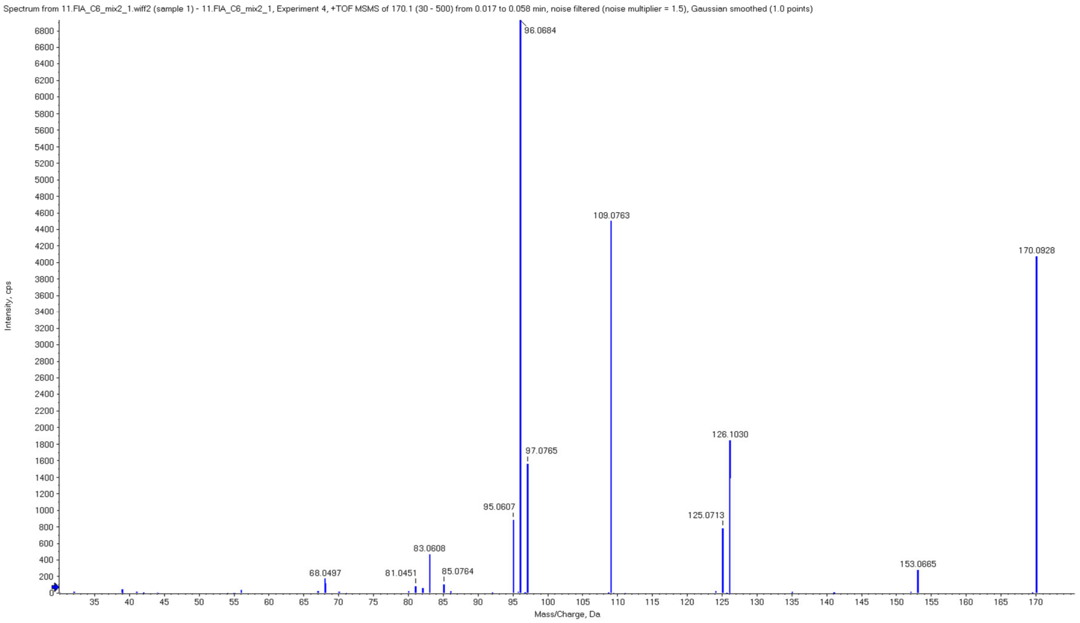

Mass/Charge (Da)	Height	Intensity Relative to Most Abundant Product Ion (%)
68.04966	175.9052	2.54
81.04515	85.11393	1.23
83.06081	472.9107	6.83
85.07638	106.4177	1.54
95.0607	882.3759	12.74
96.06844	6928.622	100.00
97.07649	1560.458	22.52
109.0763	4503.946	65.00
125.0713	782.7315	11.30
126.103	1849.442	26.69
153.0665	280.3756	4.05

NMMA (Q1 = 189.1)

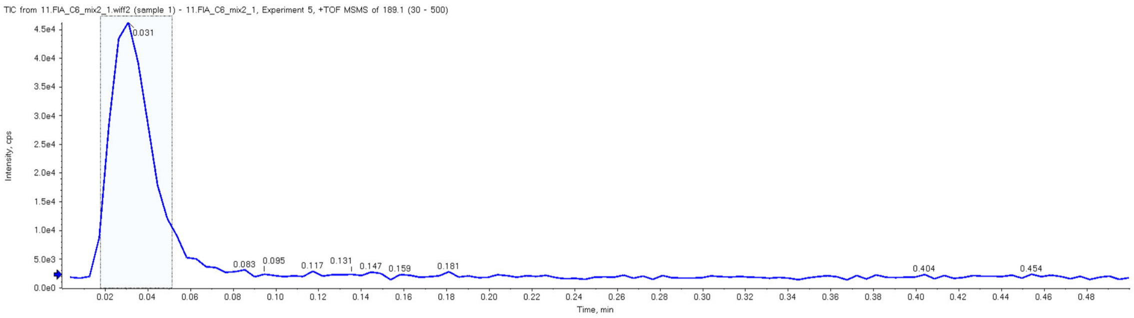

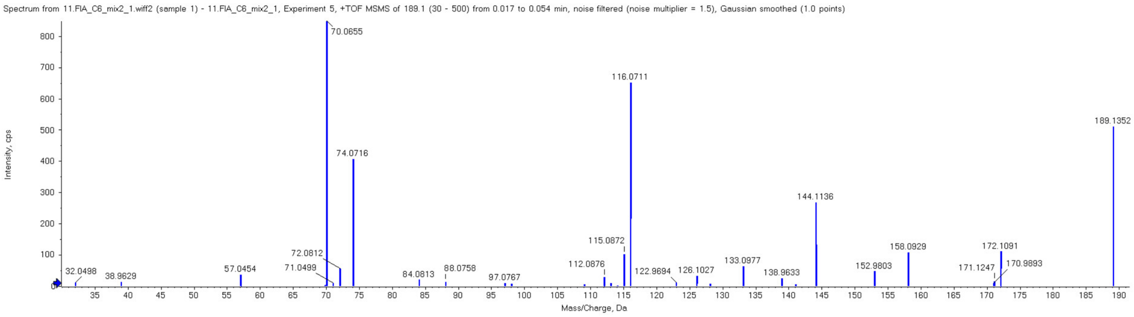

Mass/Charge (Da)	Height	Intensity Relative to Most Abundant Product Ion (%)
32.04979	12.12997	1.43
38.9629	14.95998	1.76
57.04543	37.8854	4.46
70.06548	849.152	100.00
71.04995	10.2927	1.21
72.08117	57.0277	6.72
74.07159	408.0279	48.05
84.08135	20.98035	2.47
88.07582	13.87293	1.63
97.07665	9.795668	1.15
112.0876	28.53977	3.36
113.0716	10.25396	1.21
115.0872	102.0867	12.02
116.0711	651.6549	76.74
122.9694	12.19983	1.44
126.0917	27.64764	3.26
126.1027	33.19124	3.91
133.0977	64.06	7.54
138.9633	25.70988	3.03
144.1136	268.8306	31.66
152.9803	47.82257	5.63
158.0929	107.9967	12.72
170.9893	9.703011	1.14
171.1247	16.35341	1.93
172.1091	111.4572	13.13

SDMA (Q1 = 203.2 *m*/*z*)

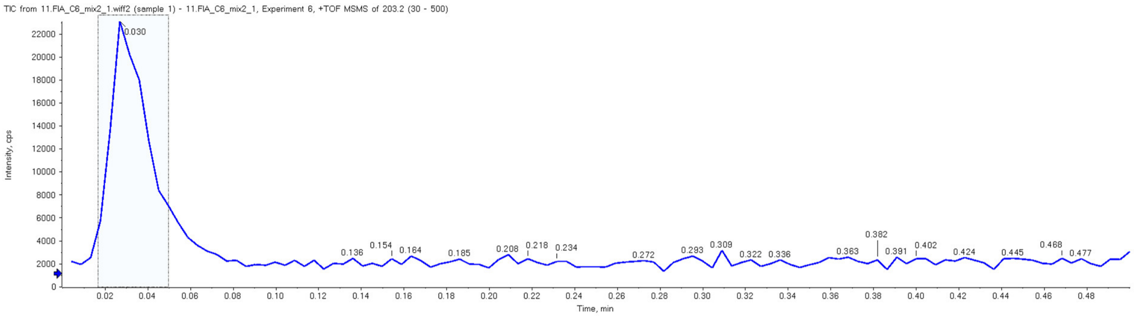

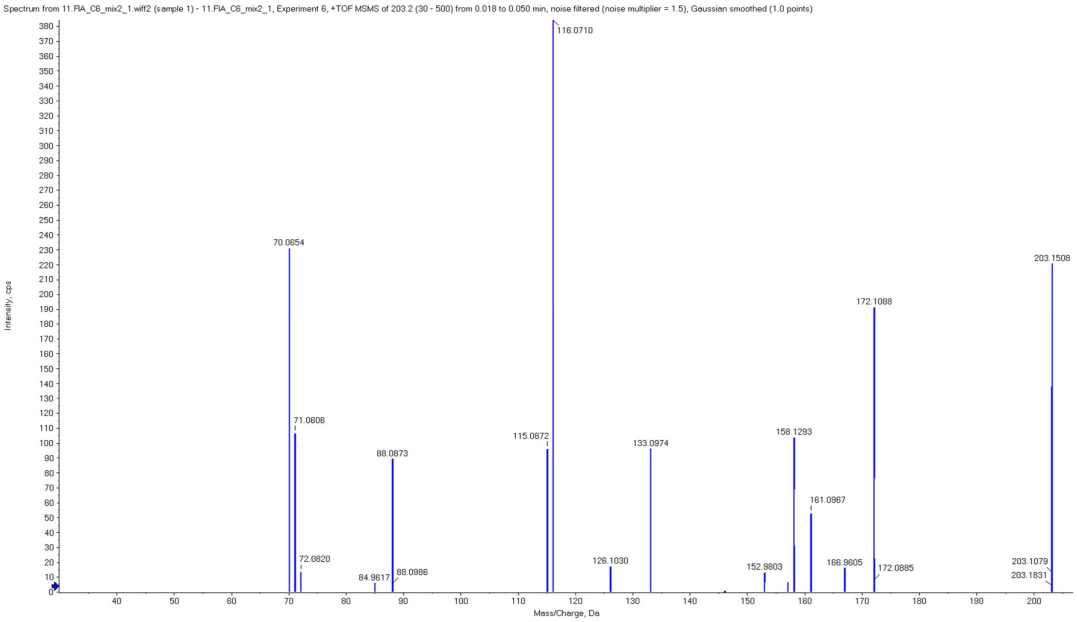

Mass/Charge (Da)	Height	Intensity Relative to Most Abundant Product Ion (%)
70.06542	231.1337	60.16
71.06063	106.5642	27.74
72.08201	13.49099	3.51
84.96171	6.073396	1.58
88.08727	89.45863	23.28
88.09864	6.005317	1.56
115.0872	96.02292	24.99
116.071	384.2013	100.00
126.103	17.34329	4.51
133.0974	96.38313	25.09
152.9803	13.21275	3.44
157.0389	6.701523	1.74
158.1293	103.6963	26.99
161.0967	52.7847	13.74
166.9605	16.25199	4.23
172.0885	8.445034	2.20
172.1088	191.4343	49.83

Table S1. The standards of the analytes with corresponding internal standard correction, supplier and C8 concentration.

Name	ChEbi ID	Internal standard correction		Standard	Supplier	Solvent	C8 (μM)
		Fractionation	HILIC				
1-Met	70958	1-Met-D3	1-Met-D3	1-methylhistidine	HMDB	H_2O	640
3-Met	70959	1-Met-D3	1-Met-D3	3-methylhistidine	HMDB	H_2O	320
Bet	17750	Val-D8	Bet-D9	Betaine hydrochloride	Sigma-Aldrich	H_2O	1200
Val	27266	Val-D8	Val-D8	L-valine	Fluka	H_2O	3200
Ile	17191	Leu-D3	Leu-D3	L-Isoleucine	Fluka	H_2O	800
Leu	15603	Leu-D3	Leu-D3	L-Leucine	Fluka	H_2O	800
hArg	27747	Arg-$^{15}N_2$	Lys-D4	L-homoarginine HCL	Sigma-Aldrich	H_2O	80
NMMA	28229	Arg-$^{15}N_2$	1-Met-D3	NG-methyl-L-Arginine Acetate Salt	Sigma-Aldrich	H_2O	16
ADMA	17929	Arg-$^{15}N_2$	1-Met-D3	NG,NG-Dimethylarginine dihydrochloride	Sigma-Aldrich	H_2O	16
SDMA	25682	Arg-$^{15}N_2$	1-Met-D3	NG,NG′-Dimethyl-L-arginine di(p-hydroxyazobenzene-p′-sulfonate)	Sigma-Aldrich	H_2O	16

Table S2. The internal standards with corresponding supplier and C4 concentration.

Name	Internal standard	Supplier	Solvent	Concentration (μM)
Val-D8	L-VALINE (D8)	Cambridge isotopes	H2O	200
Leu-D3	DL-Leucine-D3	CDN Isotopes	1M HCl	60
Lys-D4	L-LYSINE 2 HCL (4,4,5,5-D4)	Cambridge isotopes	H2O	180
Arg-$^{15}N_2$	L-Arginine-^{15}N2 hydrochloride	Cortecnet	H2O	100
Bet-D9	N-(Carboxymethyl)-N,N,N-trimethyl-d9-ammonium Chloride	CDN Isotopes	H2O	75
1-Met-D3	1-Methyl-d3-*L*-Histidine	CDN Isotopes	H2O	80

Table S3. Clinical variables of ten healthy male subjects.

Gender	Age	Race	BMI	Fasted	Smoker
Male	33	Hispanic	31	YES	NO
Male	56	African American	35	NO	NO
Male	61	Hispanic	20	NO	YES
Male	24	Hispanic	31	NO	YES
Male	33	Hispanic	30	NO	NO
Male	56	African American	33	NO	YES
Male	34	African American	27	NO	YES
Male	56	African American	41	YES	NO
Male	54	Hispanic	30	NO	NO
Male	32	Hispanic	31	NO	YES

Table S4. SWATH window sizes for the fractionation method.

SWATH windows	Variable SWATH		Fixed SWATH	
	Start Mass	Stop Mass	Start Mass	Stop Mass
1	74.5	105.5	75	101
2	104.5	132.7	100	126
3	131.7	156.2	125	151
4	155.2	178.2	150	176
5	177.2	199.6	175	201
6	198.6	221.6	200	226
7	220.6	248.8	225	250

Table S5. SWATH window sizes for the HILIC method.

SWATH windows	Variable SWATH		Fixed SWATH	
	Start Mass	Stop Mass	Start Mass	Stop Mass
1	74.5	80.5	75	81
2	79.5	86.4	80	87
3	85.4	93	86	93
4	92	99.6	92	99
5	98.6	106.9	98	105
6	105.9	113.6	104	111
7	112.6	120.9	110	117
8	119.9	127.5	116	123
9	126.5	134.1	122	129
10	133.1	140.8	128	135
11	139.8	145.9	134	141
12	144.9	151.8	140	147
13	150.8	157.7	146	153
14	156.7	163.5	152	159
15	162.5	168.7	158	165
16	167.7	174.6	164	171
17	173.6	179.7	170	177
18	178.7	185.6	176	183
19	184.6	190.7	182	189
20	189.7	195.9	188	195
21	194.9	201.8	194	201
22	200.8	206.9	200	207
23	205.9	211.3	206	213
24	210.3	216.5	212	219
25	215.5	221.6	218	225
26	220.6	227.5	224	231
27	226.5	232.6	230	237
28	231.6	237.8	236	243
29	236.8	242.9	242	249
30	241.9	248.8	248	250

Table S6. The quantification accuracy of structural isomers in comparison with a MRM^HR^. Values between 85-115% are indicated in green. Values between 115-120% are indicated in yellow. Values outside these ranges are indicated in red. Compounds that could not be quantified due to an insufficient linearity (<0.99), high variability (>15%) or integration problems (peak overlap or too high baseline) are indicated by the zero values.

Analyte	Product Ions (*m/z*)	Variable SWATH (%)		Fixed SWATH (%)		MS^ALL^ (%)	
		Fractionation	*HILIC*	*Fractionation*	*HILIC*	*Fractionation*	*HILIC*
ADMA	46.0651	88	96	0	102	0	0
	112.0873	0	119	0	121	0	0
	114.1028	233	111	0	119	0	0
SDMA	126.1028	0	86	0	94	183	0
	172.1081	107	101	96	110	106	0
hArg	60.0560	109	122	0	117	0	0
	84.0810	0	112	0	91	0	0
	85.0651	191	0	389	126	271	0
	86.0967	0	99	0	102	0	0
	127.0870	0	141	0	159	0	0
	130.0866	0	104	0	95	0	0
	147.1133	0	95	0	0	0	0
	155.0818	0	89	0	140	0	0
NMMA	115.0870	739	136	6383	226	1944	0
	158.0926	0	111	0	0	0	0
Bet	58.0655	106	195	91	0	150	0
	59.0733	0	226	114	0	0	0
	102.0550	0	102	0	0	0	358
	117.7023	131	124	103	134	136	124
Val	55.0544	98	112	93	115	99	104
	57.0582	170	91	167	91	184	101
	72.0811	0	291	148	258	142	0
1-Met	124.0868	157	113	0	109	0	0
3-Met	85.0765	338	109	288	103	0	0
	95.0608	0	96	2120	97	1723	0
	126.1029	97	99	97	89	193	89
Ile	69.0704	106	112	113	99	129	90
Leu	43.0544	144	110	148	117	171	168

Table S7. Correlation and accuracy of the quantification values of 10 structural isomers in 10 volunteers.

Analyte	Product Ions (*m/z*)	Correlation (R^2)	Accuracy (%)
ADMA	46.0651	0.95	104
	112.0873	0.91	104
SDMA	126.1028	0.98	96
	172.1081	0.98	108
hArg	84.0810	0.98	103
	86.0967	0.95	103
	130.0866	0.94	100
	147.1133	0.91	113
	155.0818	0.90	101
NMMA	158.0926	0.91	99
Bet	102.0550	0.86	157
Val	55.0544	0.95	104
	57.0582	0.93	110
1-Met	124.0868	1.00	108
3-Met	85.0765	0.99	105
	95.0608	0.99	105
	126.1029	1.00	104
Ile	69.0704	0.94	105
Leu	43.0544	1.00	97
